# Points to consider in seeking biosafety approval for research, testing, and environmental release of experimental genetically modified biocontrol products during research and development

**DOI:** 10.1007/s11248-022-00311-z

**Published:** 2022-10-04

**Authors:** W. K. Tonui, V. Ahuja, C. J. Beech, J. B. Connolly, B. Dass, D. C. M. Glandorf, S. James, J. N. Muchiri, C. F. Mugoya, E. A. Okoree, H. Quemada, J. Romeis

**Affiliations:** 1 Environmental Health Safety Consultancy Ltd., Office 10D, Sifa Towers, Lenana/ Cotton Avenue Junction, Kilimani, Nairobi, Kenya; 2 Biotech Consortium India Limited, New Delhi, India; 3 Cambea Consulting Ltd, Reading, UK; 4 Imperial College London, London, UK; 5 Foundation for the National Institutes of Health, North Bethesda, MD USA; 6 GMO Office, National Institute of Public Health and the Environment, Bilthoven, The Netherlands; 7 Foundation for the National Institutes of Health, North Bethesda, MD USA; 8 National Biosafety Authority, Nairobi, Kenya; 9 Target Malaria, Kampala, Uganda; 10 National Biosafety Authority, Accra, Ghana; 11 Western Michigan University, Kalamazoo, MI USA; 12 Research Division Agroecology and Environment, Agroscope, Zurich, Switzerland

**Keywords:** Genetic modification, Biological control, Risk assessment, Regulation, Biosafety

## Abstract

Novel genetically modified biological control products (referred to as “GM biocontrol products”) are being considered to address a range of complex problems in public health, conservation, and agriculture, including preventing the transmission of vector-borne parasitic and viral diseases as well as the spread of invasive plant and animal species. These interventions involve release of genetically modified organisms (GMOs) into the environment, sometimes with intentional dissemination of the modification within the local population of the targeted species, which presents new challenges and opportunities for regulatory review and decision-making. Practices developed for GMOs, primarily applied to date for GM crops may need to be adapted to accommodate different types of organisms, such as insects, and different technologies, such as gene drive. Developers of new GM biocontrol products would benefit from an early understanding of safety data and information that are likely to be required within the regulatory dossier for regulatory evaluation and decision making. Here a generalizable tool drawing from existing GM crop dossier requirements, forms, and relevant experience is proposed to assist researchers and developers organize and plan their research and trialing. This tool requires considering specifics of each investigational product, their intended use, and country specific requirements at various phases of potential product development, from laboratory research through contained field testing and experimental release into the environment. This may also be helpful to risk assessors and regulators in supporting their systematic and rigorous evaluation of new biocontrol products.

## Introduction

Biological control (biocontrol) is a long-standing method of pest management. In classical biocontrol, the agents typically are species-specific natural enemies of the target pest species, *e.g*., living predators, parasitoids, competitors, or pathogens, which are released into the environment to reduce and control the pest population by debilitating, competing, or killing it (Huffaker et al. 1976; Dent and Binks 2020). Another biocontrol approach termed Sterile Insect Technique (SIT) that has been applied to pest insects with established success (Knipling 1955; Van der Vloedt 1991; Dame et al. 2009; World Health Organization and International Atomic Energy Agency 2020) involves systematic, inundative, area-wide release of radiation or chemically-sterilized male insects into a wild, local insect population in a defined area to reduce its overall reproductive capacity, resulting in autocidal population suppression (Hendrichs 2000). SIT could be considered genetic biocontrol as the irradiation or chemicals alters the genetic makeup of the organism. Genetic biocontrol can also utilize a living genetically modified (GM) agent, typically a product of modern biotechnology, to control the pest population. For example, molecular biological methods have been applied to introduce specific traits into pest or vector insect species that improve the efficiency of SIT for reducing insect-generated damage to crops or animal and human disease (Alphey and Bonsall 2018). Gene drives, which promote the inheritance of certain genes across generations at a frequency higher than expected on the basis of Mendelian inheritance, are being engineered and are among the latest GM biocontrol technologies to be explored (Alphey 2014; Alphey et al. 2020). Engineered gene drive organisms intended to reduce or modify populations of disease vectors, pest populations, and invasive species are being examined for their potential to provide solutions for intractable problems in public health, agriculture, and conservation (Teem et al. 2020).

Development of disease or pest control tools using new genetic technologies, such as engineered gene drives, have raised questions about the adequacy of existing regulatory paradigms (National Academies of Sciences and Medicine 2016; Naegeli et al. 2020). The Competent Authority (CA) is the national regulatory body charged with permitting and determining the terms and conditions under which development, testing and use of GM organisms (GMOs, also referred to as living modified organisms or LMOs) occurs, especially in countries that are Parties to the Cartagena Protocol on Biosafety (CPB) to the Convention on Biological Diversity (CBD) (Secretariat of the Convention on Biological Diversity 2000). Thus, the CA ensures implementation, administration, and compliance with relevant national statutes and regulations related to GMO biosafety. As per Article 19 of the CPB, Parties are required to designate one or more competent national authorities “*responsible for performing the administrative functions required by the Protocol*”.^1^ By extension, particularly in countries that are Parties to the CPB, GMOs such as those being developed for biocontrol will be regulated from a biosafety perspective. Typically, the pathway for biosafety approval will involve submission of a dossier containing various data and information on specifics of the GMO including a risk assessment that considers the potential for harm to human or animal health and the environment (CPB Annex III- Risk Assessment; (Secretariat of the Convention on Biological Diversity 2000)) as required by national law and implementing regulations, and consistent with the country’s international obligations. The initial step in the biosafety regulatory pathway will likely be providing requisite information to the Institutional Biosafety Committee (IBC) at the applicant organization [Sect. 7-Biosafety, (World Health Organization 2021)]. In many countries, including those that are Parties to the CPB, the IBC could be constituted through the CA.

Most countries that have experience in regulating GMOs have assessed GM crops. However, the different characteristics of GM plants versus GM insects or other animal species, as well as the range of GM systems (from sterility to self-sustaining drive containing strains) that are contemplated, may require that applicable national biosafety laws and regulations be adapted and, in some cases, revised, and that current regulatory practices likewise be reconsidered for their appropriateness to these new technologies. Concerns have been voiced that GM biocontrol agents especially those incorporating engineered gene drives, pose risks to health and the environment that are sufficiently different from any encountered to date and could be difficult to assess and manage (Simon et al. 2018; Devos et al. 2021).

A workshop convened in 2019^2^ brought together a group of international experts in regulatory science and risk assessment to consider specifically whether gene drive-modified insects for use as GM biocontrol agents in public health or agriculture present any new issues that are not addressed by existing regulatory frameworks and procedures currently used to assess regulated insects or regulated insect control products. This group agreed that safety evaluation of GM biocontrol products, including gene drive modified insects, should build on existing risk assessment paradigms. They noted that bottlenecks in the regulatory process often are caused by the presumptive amount of safety data required, and therefore it would be useful to provide researchers and developers with an early understanding of anticipated data expectations for preparation of a regulatory dossier for GM biocontrol products. As a result of these discussions, the group proposed that it would be helpful to compare existing regulatory application forms from countries that have experience with regulation of GMOs, especially GM crops, and create a draft consensus application form appropriate for GM biocontrol products.

The work reported here responds to the recommendation from the 2019 workshop. Because it was not possible to convene an in-person, follow-up workshop due to COVID-19 pandemic related travel restrictions, the process of identifying consensus application questions was taken up as an online collaboration. Building upon their own experience as well as relevant risk assessment guidance from multiple national and international organizations (reviewed in (World Health Organization 2021)), 30 international experts were invited to participate of which 18 worked over a two-year period to provide the recommendations presented here. After the initial draft consensus document was distilled from comparison of GMO (crop) application forms from 10 countries, it underwent two rounds of online review and revision followed by online discussions of any issue for which there was a need for clarification or there were deviating views. The biosafety regulatory forms and procedural documents from Burkina Faso, Ghana, Nigeria, Kenya, South Africa, Brazil, Philippines, India, Australia, Netherlands, and the European Food Safety Agency (EFSA) were consulted. These countries and their relevant agencies were chosen based on their experience using their country laws and regulations to approve GM crops and the expectation that GM approaches for insect vector control would likely be undertaken initially in African countries that are Parties to CBD and the CPB.

GM biocontrol products may be developed in-country or imported into the country under an import permit and appropriate oversight. Research, testing, and use activities could include, but are not limited to contained use in-country for research or experimental^3^ use; export; surveillance, monitoring, or testing of field caught specimens, and controlled release testing. The recommendations presented here cover anticipated application requirements across various phases of GM biocontrol product development, from laboratory research through contained field testing (*e.g.*, field cages) and experimental controlled release into the environment. While the steps identified here originated from discussions on the regulation of GM biocontrol products for insect disease vectors and pests, they should be informative to those conducting research or developing other types of GM biocontrol products. They could serve as a basis for researchers and developers to identify and prioritize data and information that will be most important to inform safety and risk assessment and that therefore may be expected to be part of a regulatory dossier. Considering that many countries are yet to develop specific laws or guidance for GM biocontrol products, these recommendations on fundamental information to underpin biosafety evaluation may also be of interest to risk assessors, regulators, and policy makers.

## Anticipated application considerations

The overall experimental development of candidate GM biocontrol agents leading to the commercial release/placing on the market of a registered product (if approved) may follow the phased approach recommended for GM mosquitoes by the World Health Organization (World Health Organization 2014, 2021). This begins with testing under physical containment. The level of containment is reduced, and the scale of release is increased gradually, only if decision makers are satisfied that the safety evaluation of the earlier steps in terms of protection of human and animal health and the environment indicates an acceptable level of risk to benefit justifying taking the next step (James et al. 2018). Case-by-case risk assessment and decision-making will be based upon country-specific requirements of data about the characteristics and behavior of the GM biocontrol product, its intended use, the potential receiving environment, and in some cases socioeconomic considerations. The nature of the information required, and level of detail will also vary according to the testing phase.

### Project planning and preparation

For research or testing of a candidate GM biocontrol product to take place in a country, developers will need to consider particular aspects of the research and regulatory environment of the host country and the capacity of the host institution and any field sites prior to initiating the application process (Table 1). Prior to beginning the project the researcher or developer should undertake or commission a legal analysis of the relevant legislation in the specific country or countries (if work will be done on a regional basis) along with reviewing information as in Table 1 as this will help them determine what regulatory requirements they must address and what capacity building or support might be required to ensure that the research environment for product development will be appropriate.

**Table 1:**
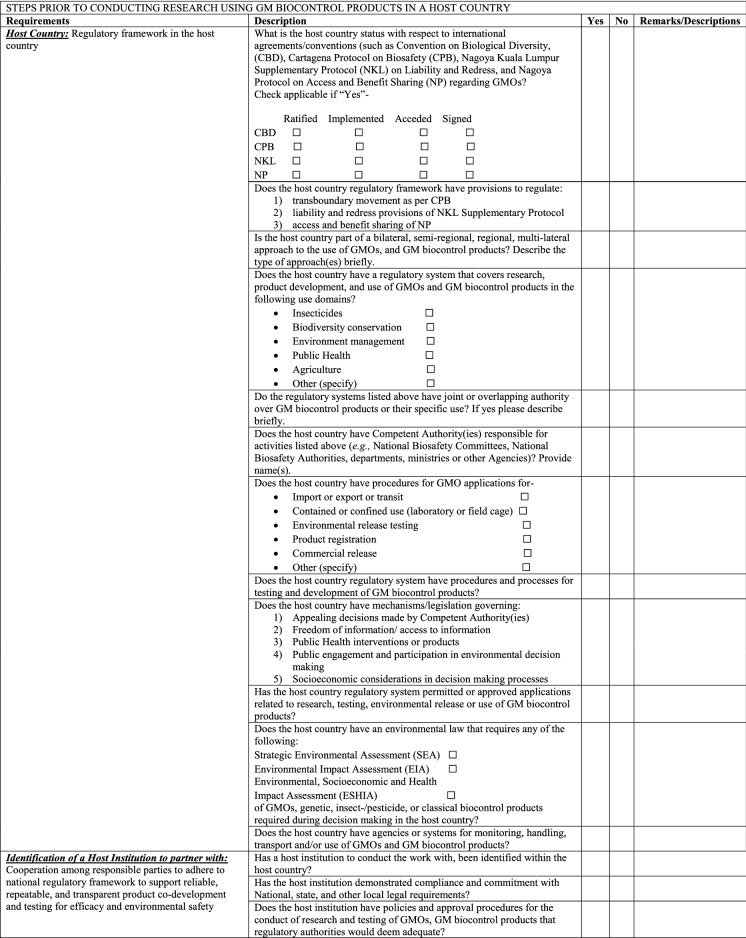

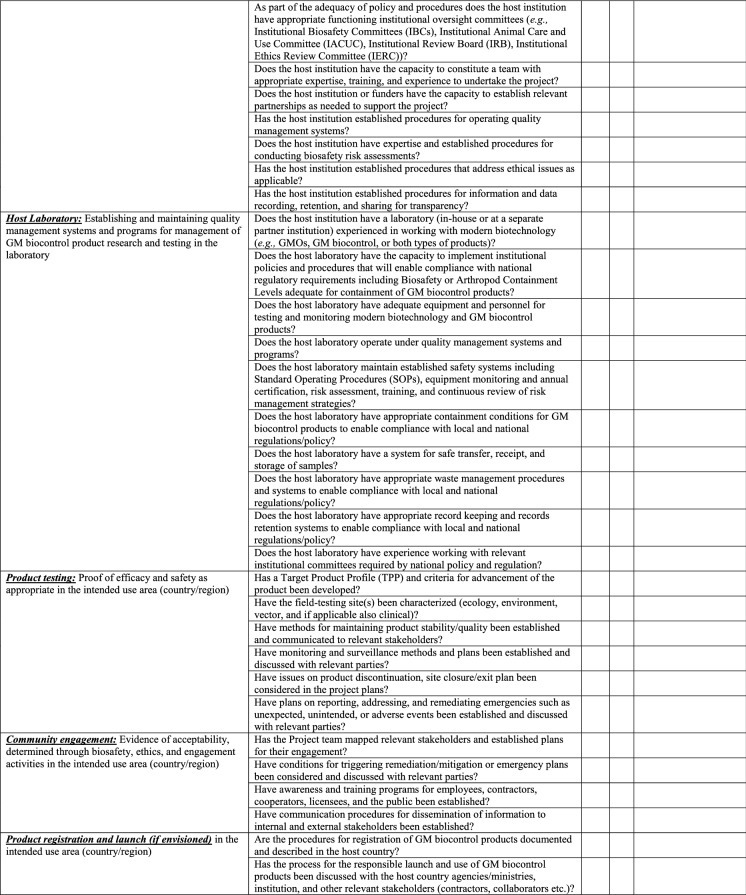
General checklist for researchers and developers on preparations for initiation of research in a host country

### Institutional biosafety committee (IBC)

Institutional Biosafety Committees (IBCs) are established by the research organization and registered by the CA to provide local review and oversight of nearly all types of research involving recombinant or synthetic nucleic acid molecules. In some cases, institutions may decide to expand the role of their IBC to include review of experiments involving other agents that are potentially infectious or hazardous. Researchers and developers can expect to interact with the IBC, if in place, prior to preparing and submitting a regulatory application to the appropriate CA at each stage of their project from import to contained use^4^ and contained field testing release^5^ to controlled release.^6^ The IBC will review the same information and dossier that will eventually be shared with the CA for their review and decision-making process prior to any activity involving the GMO (i.e., contained use, contained or controlled release, registration, and placement on the market) taking place. IBCs consider all elements of biosafety along the development pathway of GM biocontrol products that are under the institution’s control (Table 2). For example, for GM biocontrol products this would include determination of the suitability of physical and biological containment and control procedures (American Committee of Medical Entomology 2022), safety to workers in the facility, that the research is conducted to accepted standards of practice and there were appropriately trained staff to conduct research (broadly covered under "Host Laboratory: Establishing and maintaining quality management systems and programs for management of genetic or biocontrol products research and testing" in Table 1). The IBC also notifies the researcher of the result of the review and when appropriate its approval and terms and conditions of approval. Amendments may be required to the conduct of the research, or the facility in order to meet the terms and conditions of the IBC. Depending on the nature of the work and local and national legal requirements, researchers may be able to start work after the study has been approved by the IBC, while in other jurisdictions the IBC approval may be the start of the national regulatory process. In the latter case research can commence only after national regulatory review has been undertaken and approval given. Modifications to the research plans are made via study amendment requests that require approval by the IBC prior to being initiated. Researchers are also required to notify the IBC in case of any violations, accidents, exposures, loss of containment or other adverse event occurring during the conduct of the research so that previously established correctional or contingency plans can be implemented.

**Table 2:**
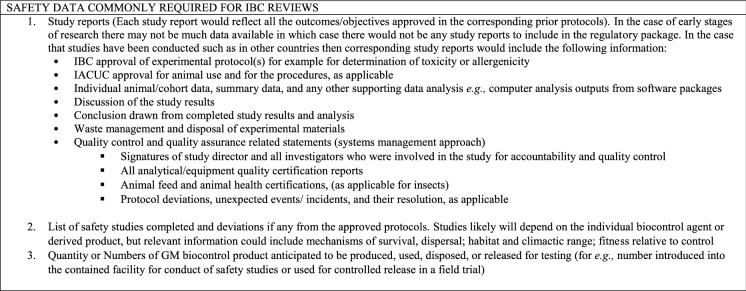
Anticipated informational requirements by IBC for a dossier to be submitted to the CA

Information in Table 2 will be in the dossier and shared first with the IBC and eventually with the CA for their respective review and decision-making process. Typically, these are conclusions and the supporting data and information from experiments (*e.g.*, toxicity studies) that have been conducted to support the various biosafety considerations that the CA would need to evaluate before making a decision related to the researcher or developer’s request.

### General project information required for CA review

Regulatory application forms typically will require a brief initial description of the product to be evaluated, the purpose of the project, the intended use covered by the application, the location(s) of the project, and the involved entities and points of contact (Table 3). This information allows developers to provide context for the CA to establish its internal procedures in response to the application as well as have a point of contact that is legally responsible for the research and its conduct.

**Table 3:**
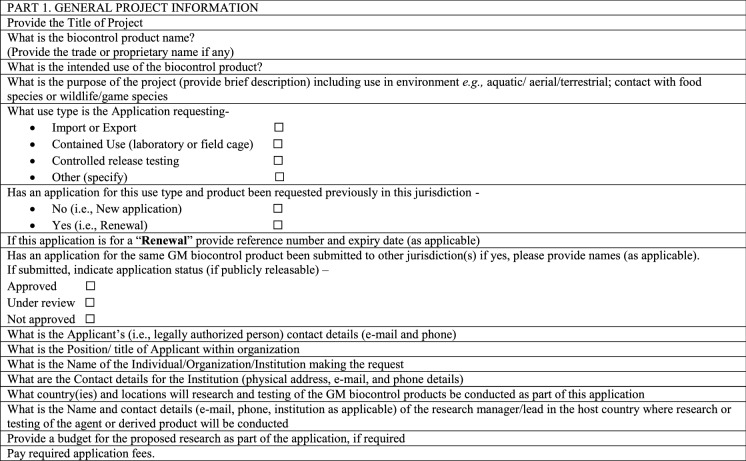
Considerations for general information on a research project involving a GM biocontrol product

### Laboratory and contained use- experimental research

The initial application to the CA is likely to be a request for permission to conduct research on the GM biocontrol product under containment in a laboratory or other physically confined environment. For example, in the case of insects, containment facilities may include an indoor insectary or large population cage. Testing of insects in large outdoor cages (contained field testing) also is possible. Potential for local establishment of the modification in the wild population will be a containment consideration for GM biocontrol products containing a gene drive modification especially for research facilities in areas with conspecifics that can interbreed (James et al. 2018; World Health Organization 2021). If the product was developed in another country, this application also may encompass a request for importation. There may also be a separate application for constructing an appropriate containment facility for rearing and research use of the GM biocontrol products prior to import or in-country development of the products, including a requirement for inspection and certification of the new facility. Details related to applications for facility construction, certification, or registration are not described in this paper. No research in the described contained facility using the particular GM biocontrol product can be undertaken until the CA has approved the application and provided the corresponding permit and terms and conditions.

Table 4 details information anticipated to be required in the application to obtain a permit for contained use from the CA in most countries. This will include general information on the research plan and site, specific information on safety data, the GM biocontrol product, and its intended use, as well as risk assessment and risk management plans.

**Table 4:**
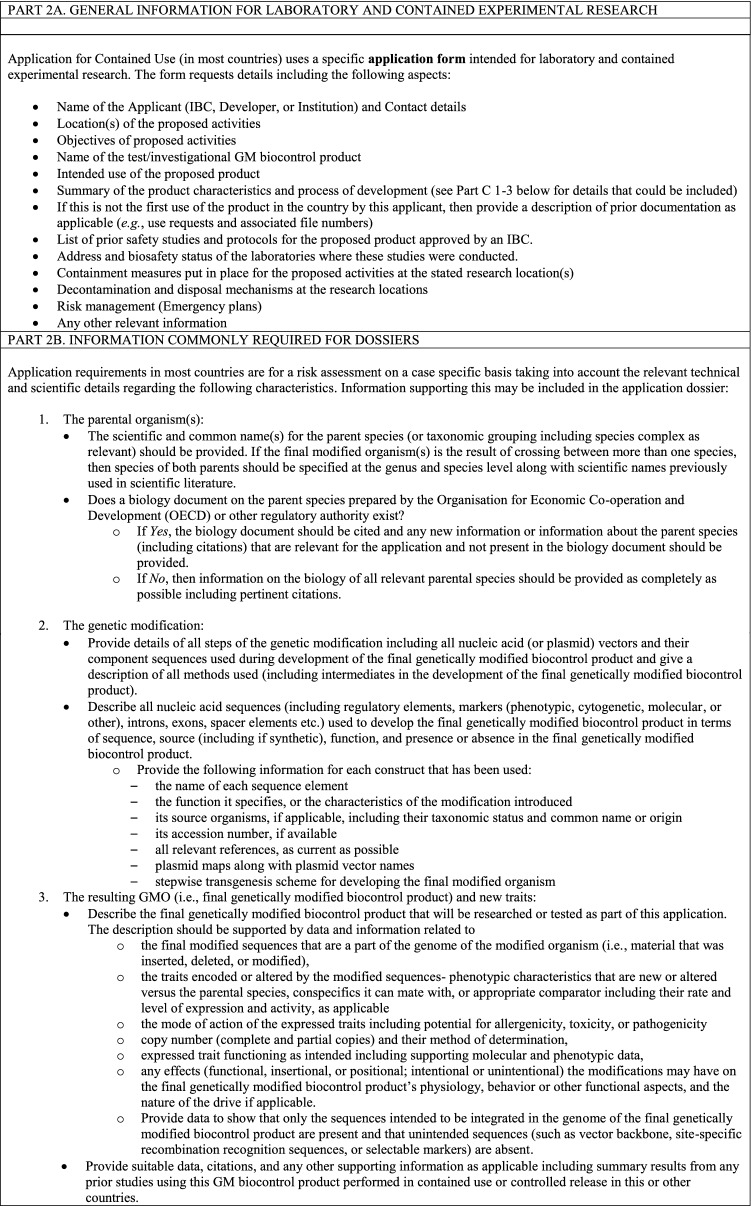

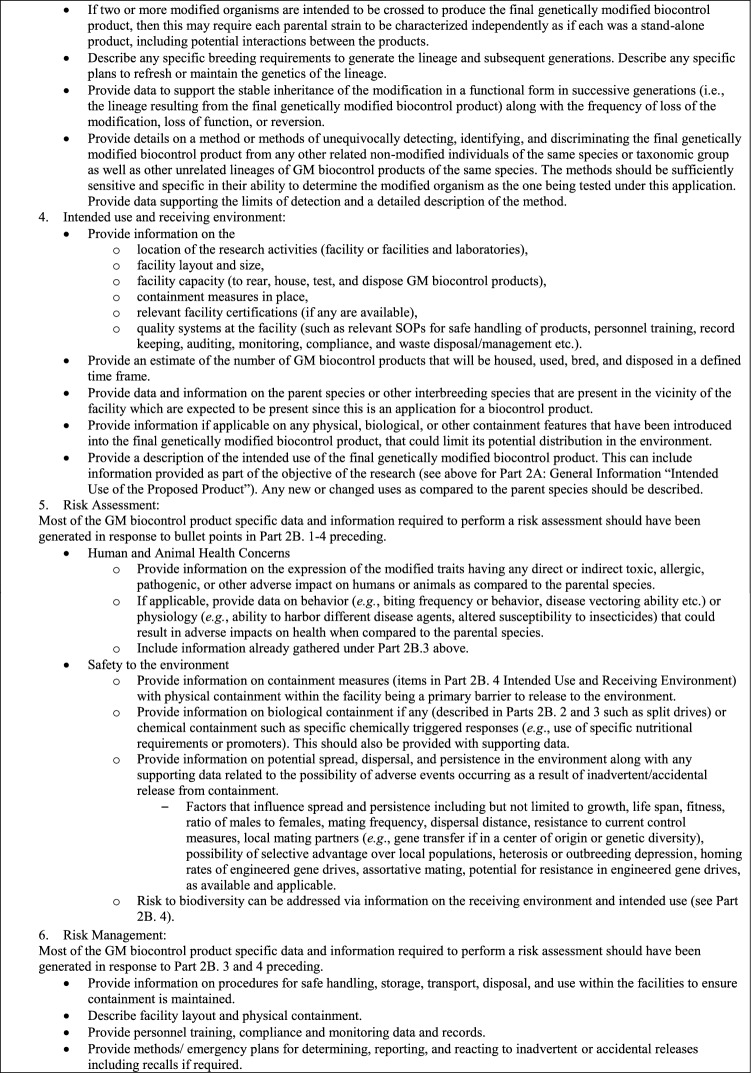
Considerations for a permit for laboratory and contained experimental research of GM biocontrol products in a regulatory application to the CA

### Environmental release for GM biocontrol product testing

An application for a permit to release a GM biocontrol product into the environment will contain many of the same elements as described under considerations for contained use (Table 4). However, the information on the intended use and receiving environment will focus on specifics of the proposed release site and plan. Information on potential spread, dispersal, and persistence of the GM biocontrol product in the environment will take on increasing relevance and may include both data collected from prior contained use studies as well as predictions from computer simulation modelling. No release of investigational, candidate GM biocontrol products should be carried out unless the consent of the CA has been obtained (Table 5).

**Table 5:**
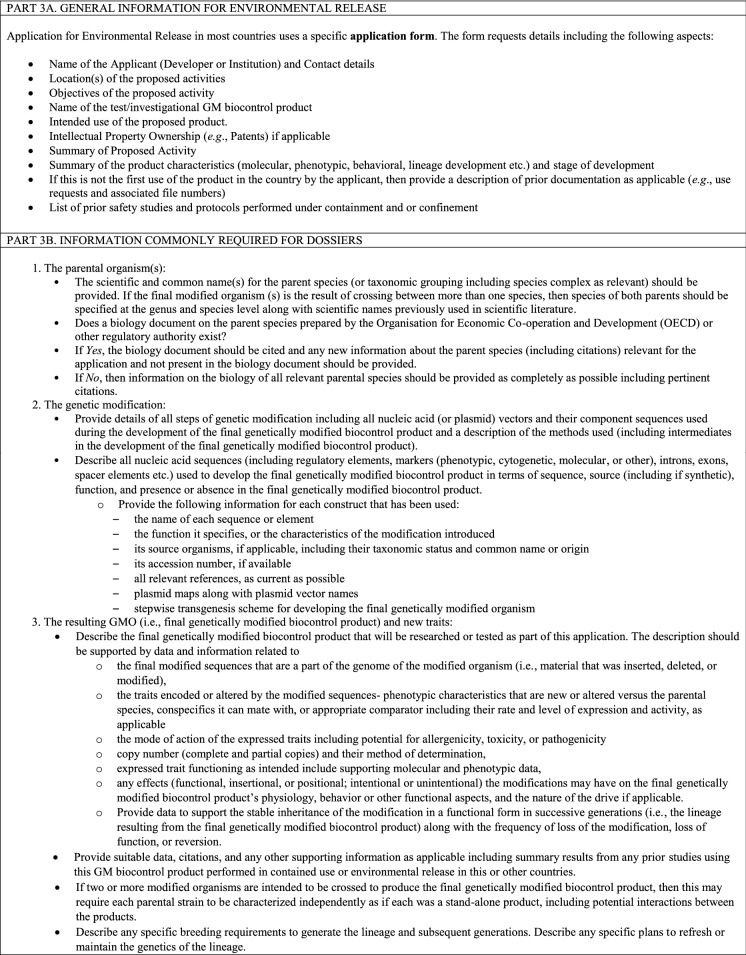

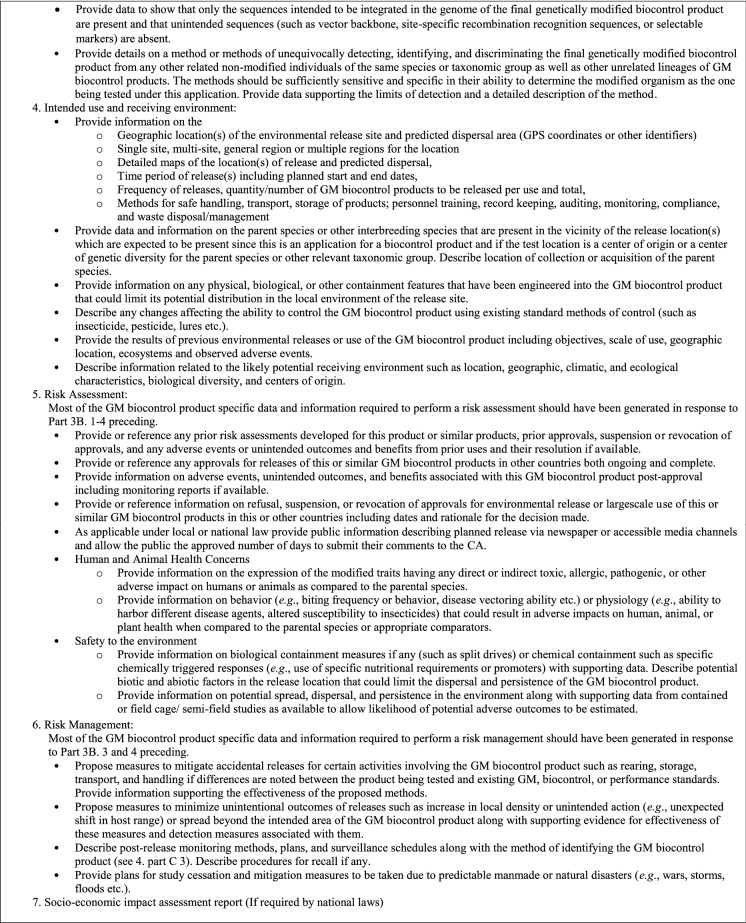
Considerations for a permit for environmental release of GM biocontrol products

## Additional information on administrative process

While this section does not include application related information per se it is included to provide a complete description of the application and regulatory review process to allow developers of GM biocontrol products to have a better understanding of the entire process.

### Dossier evaluation and permitting

Within a specified period of receipt, as prescribed by existing country-specific regulation or law, the CA will screen for completeness, including payment of the application fee and other accompanying information that may be requested by the CA to complete the file. Based on country law, the CA may be required to inform the public of the receipt of such an application including providing a summary via print, electronic, or other media.

If the CA determines the application file to be.

- “Incomplete”- this status will require the regulatory clock to be stopped and additional information will be requested from the applicant.
- “Complete”- this status will be acknowledged by the CA and it will initiate risk assessment reviews.

Common steps followed during decision making:

1. The complete application as prescribed in country laws and procedures will be provided by the CA to appointed experts and appropriate agencies for them to conduct a risk assessment. The results of this assessment will be documented in a report.
2. The complete application may be sent to other experts or public institutions that the CA deems necessary to inform the decision-making process.
3. The CA may, in addition to the comments received from other experts or public institutions, hold public hearings or consultations to obtain further comments and input that will assist in the review or processing of the Application.
4. The Applicant may be asked to provide additional information in relation to comments received from the public or the experts that were consulted as prescribed in national laws.
5. The CA communicates via a decision document whether an authorization is granted, and a permit is issued within a time period prescribed by country law, after receipt of a complete application has been confirmed.
6. The decision will be made based on:
  The complete information provided in the Application file;
  The risk assessment report taking into account risks to human health, animal health, biodiversity, and the environment;
  Relevant comments submitted by the CA, expert reviewers, other institutions, and the public;
  Socio-economic considerations determined by the CA (which may include the impact on sustainable development and the social impact of the GM biocontrol product as relevant based on country law and regulation).
7. If an authorization is granted, the CA will indicate the terms and conditions including monitoring and reporting requirements.
8. The CA may place a copy of their decision(s) which include a risk assessment report, validity period, and terms and conditions of approval in the public domain; either in a national publication on government business or on the biosafety clearing house of the Cartagena Protocol (if a Party) in the case of field release or general release.

An applicant should be able to withdraw his/her dossier at any stage of the administrative procedures, the administrative procedure should come to an end when a dossier is withdrawn. Upon withdrawal, the CA must respect the confidentiality of the information supplied.

### Permit conditions

Once issued, a permit is a legally binding instrument and penalties provided in the national law may apply for breach of approval/permit conditions. The legally binding nature of the permit conveys that the studies must be conducted as specified in the application file to which the permit is linked. The CA is likely to inspect the conduct of the trial to determine compliance with the permit conditions. Non-compliance could result in penalties and fines as mandated in national law. Permit holders are recommended to keep a correspondence file and records of documents that have been exchanged with the CA as an administrative record. This may be periodically inspected by the CA. If permit holders wish to alter the approved research protocol, prior notification and approval by CA is required.

Permit conditions may require that a permit holder must:

1. Provide details of any adverse effect to health or the environment that becomes evident during the release, as per the timeframe specified in the permit or national regulations/law;
2. Report(s) in relation to the range of permitted activities as per terms and conditions;
3. Provide periodic reports of post-release monitoring activities and results;
4. Provide a final report at the end of the monitoring period.

Country specific regulations and/or the CA will determine the procedures for amending protocols and studies, if necessary, after a permit has been issued. In the event of any modification of, or unintended change to, the permitted activity that could have consequences with regard to risks for human health and the environment after the CA has given its written consent, or if new information has become available on such risks either while the dossier is being examined by the CA or after that authority has given its written consent, the applicant must immediately: take the measures necessary to protect human health and the environment; inform the CA in advance of any modification or as soon as the unintended change is known, or the new information is available; and revise the measures specified in the dossier in consultation with the CA.

### Reporting

Following the release of a GM biocontrol product, the applicant shall ensure that monitoring and reporting on it are carried out according to the conditions specified in the decision document provided by the CA. The reports of this monitoring shall be submitted to the CA. The CA which received the original application may adapt the monitoring plan after the first monitoring period based on the results of the monitoring conducted and reported as previously specified.

After a permitted activity (*e.g.*, contained use research study, confined use, or environmental release has been completed, developers can expect that a final report will be required to be submitted to the IBC and CA. Components of the report are likely to include:

1. Project details:
  Name of Institution
  Regulatory Permit Number
  Title and purpose of experimental release into the environment
  Person legally in charge and contact information.
2. If the activity involved environmental releases, details on releases:
  Number or quantity of GM biocontrol products released in total and per release (if multiple releases)
  Number of releases conducted
  Location of initial release(s)
  Dates of first and last release.
3. Biosafety measures adopted and whether such measures were in line with the CA’s approval terms and conditions.
  Monitoring methods used and whether such measures were in line with the CA’s approval terms and conditions.
  Information on survival of the GM biocontrol product within the dispersal area after completion of the experiment.
  Information on the persistence of genetic elements (background genome) in instances where organisms can hybridize with wild type counterparts; presence of relevant traits (*e.g*., insecticide resistance in mosquitoes), evaluated in the local population for persistence and influence on native phenotypes over several generations. (This could be unnecessary when hybrid lethality is demonstrated at 100% penetrance.)
4. Summary of results achieved, and indication of attainment of the objectives of the planned release.
5. Report on any unexpected/inadvertent/adverse effects recorded during the planned release; and
6. Information on whether the CA supervised the experimental release, including a copy of the Supervision Report and Violation Record, as applicable.

## Discussion

This report describes steps for seeking biosafety approval for investigational, candidate GM biocontrol products at the institutional and national level during their development and testing. The recommendations provide a tool for organizing and planning on a case-by-case basis the information to be included in dossiers required for safety evaluation and regulatory approval for laboratory research, contained field testing, and controlled release of such GM biocontrol products. Although originally envisaged for GM including gene drive modified insect biocontrol products, these recommendations also may be informative for other types of genetic biocontrol products. This paper does not describe steps for registering for commercial use or placing GM biocontrol products on the market.

While these recommendations focus on biosafety approval through an IBC and CA, multiple national authorities may be relevant for some GM biocontrol products. For example, health ministries will have particular interest in those products aimed at disease control, and some countries have environmental laws that require strategic environmental assessments (SEA),^7^ environmental impact assessment (EIA),^8^ or environmental, socioeconomic and health impact assessment (ESHIA),^9^ which considers potential positive and negative health, environmental, economic, and cultural impacts. Thus, an important initial step in project planning is ensuring awareness of all relevant laws and consulting the appropriate authorities/ministries prior to commencing work in the particular country.

Some countries have experience with biosafety regulation of GMOs, including GM insects. Participants in the 2019 workshop that led to the development of these recommendations agreed that safety evaluation of GM biocontrol products, including those containing gene drive modifications, should build on existing biosafety and risk assessment paradigms used in these countries. More recently, the GMO Panel of the European Food Safety Authority (EFSA 2020) also concluded that risk assessment of gene drive-modified insects can build on existing frameworks for GM insects and be informed by experience releasing insects for biological and genetic disease vector/pest control. Thus, the recommendations provided here are derived from and extend existing biosafety application forms whose utility has been validated through experience with GM insects and other GMOs. They strive to anticipate information that will be relevant to safety evaluation for a wide range of GM biocontrol technologies including engineered gene drive containing organisms, drawing on relevant guidance (National Academies of Sciences and Medicine 2016; Secretariat of the Convention on Biological diversity 2020; Naegeli et al. 2020; World Health Organization 2021).

These recommendations consider information required for safety evaluation of GM biocontrol products at all stages of development. Much of the anticipated information in applications for contained use or environmental release, such as data on the parental organism and genetic modification, will be similar. However, information on the receiving environment and intended use will differ substantially among different use cases. Likewise, plans for risk assessment and risk management are expected to diverge, based on potential for spread and dispersal of the GM biocontrol product. Containment may encompass physical confinement in indoor or outdoor facilities. Environmental release may be conducted in phases, beginning with isolated small-scale testing, and building to larger scale testing under different conditions (World Health Organization 2021). It is expected that separate biosafety approval will have to be sought for each of these different phases of release, based upon changes in characteristics of the receiving environment and their influence on risk assessment and risk management planning.

Some countries still either have not fully developed their own biosafety laws and regulations, or only have familiarity with regulation of GM crops and have not considered requirements for information to be included in dossiers for contained use and field trials of GM insects or other animals. Thus, in addition to providing a planning aid for developers, the recommendations presented here, based on a compendium of relevant regulatory experience, offer useful practical guidance for countries seeking to develop regulatory requirements for GM biocontrol products. It would be particularly valuable if this helped to support harmonization of procedures and criteria for case-by-case evaluation in regions where these products are likely to be tested.

## Footnotes

1 Cartagena Protocol on Biosafety Article 19 can be accessed at https://bch.cbd.int/protocol/text/article.shtml?a=cpb-19.

2 More information about this workshop can be accessed at: https://fnih.org/our-programs/geneconvene/Strengthening-Capacity.

3 The term “experimental” as used throughout this paper refers solely to research and development related testing and use of investigational, candidate, GM biocontrol products. This could include contained, confined, as well as controlled open field release for safety or efficacy studies. This paper does not describe the types of data and information that could be required for placing a product on the market and the commercial release of registered products. As described here GM biocontrol products are not different in this sense from GM crop regulation.

4 For our purposes here “contained use” refers to any activity (such as rearing, maintenance, and housing) involving the investigational, candidate GM biocontrol organism (applies to all lifecycle stages, eggs, larvae, pupae, and adults) all occurring within a containment facility (laboratory, insectarium, rearing facility etc.) at the appropriate containment level (ACL/BSL etc.) and oversight, with removal from the facility based on prior authorized use (e.g., import, export, or disposal).

5 "Contained field testing release" as used here refers to any regulatory agency authorized or permitted caged field trial carried out in natural environmental setting of investigational, candidate GM biocontrol products for the purpose of collecting scientific data on safety and efficacy.

6 "Controlled release" as used here refers to any regulatory agency authorized or permitted experimental release of the investigational, candidate GM biocontrol product into the environment such that it can spread and disperse freely.

7 Strategic environmental assessments (SEA) is a systematic process that extends the principles of environmental impact assessment beyond the project level to the analysis of environmental effects due to proposed policies, programs, plans, and other strategic actions. The purpose of undertaking SEA is to enable public accountability by providing an overarching view of cumulative environmental and sustainability effects due to a proposed program, plan or policy to assist the decision maker. This top down approach should minimize the number and complexity of stepwise EIAs required thereby decreasing overall time and cost. Fischer, T. B. (2003). "Strategic environmental assessment in post-modern times." Environmental Impact Assessment Review **23**(2): 155–170.

8 Environmental impact assessment (EIA) is a process of systematically identifying and evaluating environmental effects of a proposed action and its reasonable alternatives. It also includes the determination of potential mitigation and management strategies that could be employed as a response if the activity were to be conducted. (Defined in https://www.iaia.org/pdf/Training/CaseStudies/Acronyms.pdf).

9 Environmental, socioeconomic and health impact assessment (ESHIA) extends the SEA paradigm to include socio-economic (such as impacts on community, social structure and stability, and quality and way of life) and health impacts of the proposed action where health is as defined by the WHO to include “social, physical and psychological well-being and not just the absence of disease.
